# Deep learning-based prediction of post-pancreaticoduodenectomy pancreatic fistula

**DOI:** 10.1038/s41598-024-51777-2

**Published:** 2024-03-01

**Authors:** Woohyung Lee, Hyo Jung Park, Hack-Jin Lee, Ki Byung Song, Dae Wook Hwang, Jae Hoon Lee, Kyongmook Lim, Yousun Ko, Hyoung Jung Kim, Kyung Won Kim, Song Cheol Kim

**Affiliations:** 1 Division of Hepatobiliary and Pancreatic Surgery, Department of Surgery, Asan Medical Center, Brain Korea21 Project, University of Ulsan College of Medicine, 88, Olympic-ro 43-gil, Songpa-gu, Seoul, 05505 Republic of Korea; 2 Department of Radiology and Research Institute of Radiology, Asan Medical Center, University of Ulsan College of Medicine, 88, Olympic-ro 43-gil, Songpa-gu, Seoul, 05505 Republic of Korea; 3 R&D Team, DoAI Inc., Seongnam-si, Gyeonggi-do Republic of Korea; 4 Department of Convergence Medicine and Radiology, Research Institute of Radiology and Institute of Biomedical Engineering, Asan Medical Center, University of Ulsan College of Medicine, Seoul, Republic of Korea

**Keywords:** Gastroenterology, Medical research, Risk factors

## Abstract

Postoperative pancreatic fistula is a life-threatening complication with an unmet need for accurate prediction. This study was aimed to develop preoperative artificial intelligence-based prediction models. Patients who underwent pancreaticoduodenectomy were enrolled and stratified into model development and validation sets by surgery between 2016 and 2017 or in 2018, respectively. Machine learning models based on clinical and body composition data, and deep learning models based on computed tomographic data, were developed, combined by ensemble voting, and final models were selected comparison with earlier model. Among the 1333 participants (training, n = 881; test, n = 452), postoperative pancreatic fistula occurred in 421 (47.8%) and 134 (31.8%) and clinically relevant postoperative pancreatic fistula occurred in 59 (6.7%) and 27 (6.0%) participants in the training and test datasets, respectively. In the test dataset, the area under the receiver operating curve [AUC (95% confidence interval)] of the selected preoperative model for predicting all and clinically relevant postoperative pancreatic fistula was 0.75 (0.71–0.80) and 0.68 (0.58–0.78). The ensemble model showed better predictive performance than the individual ML and DL models.

## Introduction

Postoperative pancreatic fistula (POPF), as major complication of pancreatectomy, increase morbidity and mortality despite several preventive measures in 10–40% of the patients^1,2^. Several risk prediction models have been developed to identify high-risk patients in the perioperative period, and the risk factors in these models include body mass index, pancreatic softness, and pancreatic duct size^3–7^. These models are characterized by simplicity and convenience for quick bedside use. In contrast, the newly discovered factors related to POPF were reported as improved modalities. For example, anthropomorphic features, including proportions of subcutaneous fat and skeletal muscle, which could represent fatty pancreas, or obesity, are potentially associated with POPF^8^. Several perioperative factors, including diabetes, neoadjuvant chemotherapy, pancreatic steatosis, and remnant pancreatic volume, were associated with POPF^9^. Earlier models focused solely on the essential factors for simplicity, and there are few reports that included the newly developed risk factors. A comprehensive model incorporating both classical factors and newly discovered factors is required.

Recently, machine learning (ML) has enabled comprehensive modeling comprising a large amount of variables. Moreover, deep learning (DL) models have facilitated analytical processing of imaging-based data^10^ that can enable various applications. Several studies have investigated POPF prediction ML or DL models established using perioperative clinical and computed tomography (CT)-based data^11–13^. However, they were limited because of their small sample sizes or higher event rates. Most studies applied specific ML models without conducting a comparison between ML models. Some studies did not include comparison with conventional models. This study aimed to establish a prediction model for POPF and generate CR-POPF models using either preoperative-only or perioperative data.

## Results

### Participant characteristics

**Figure 1:**
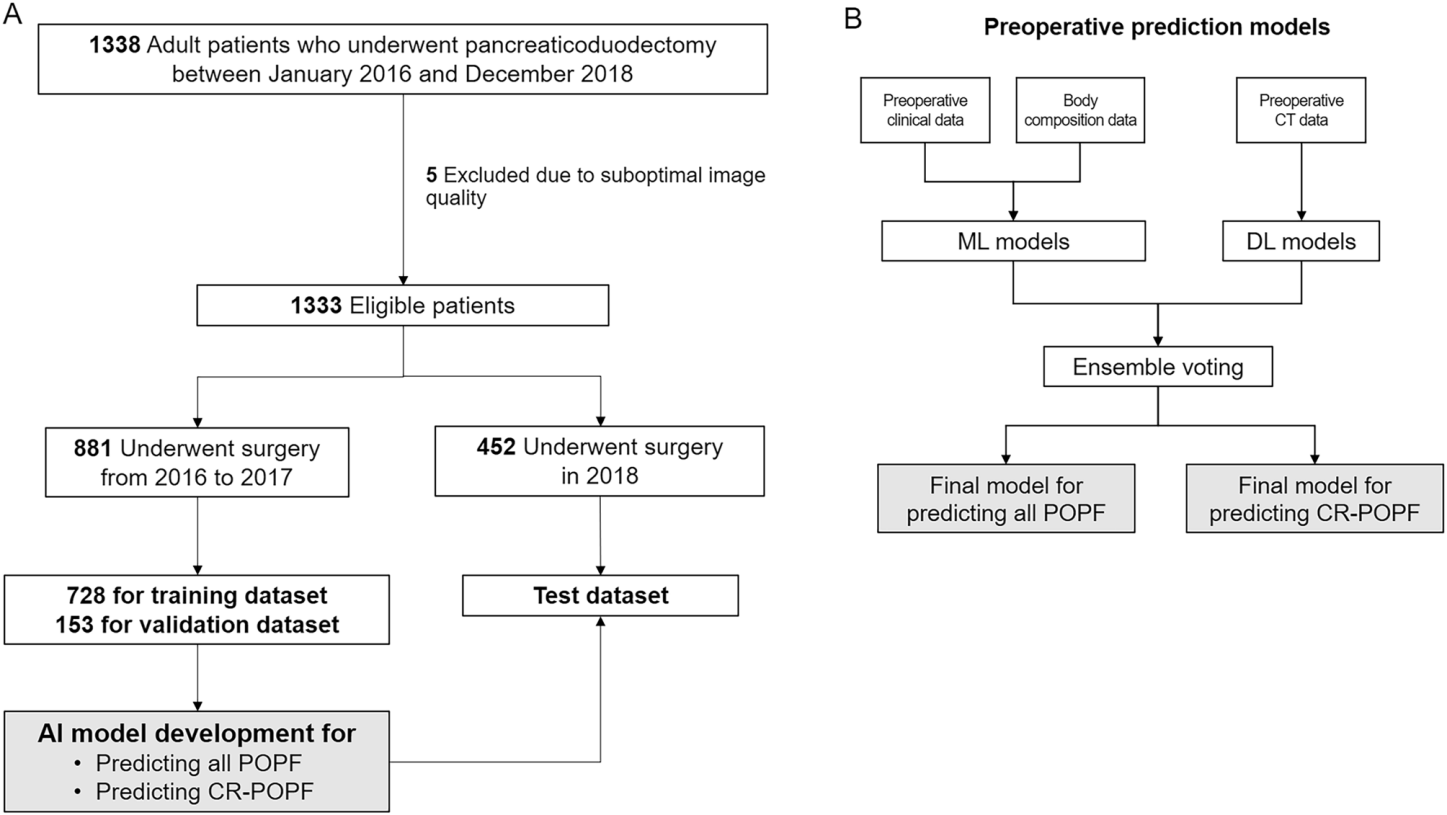
Study flowchart and schematic representation of the developed models. For training the models, data of the 881 patients who underwent pancreaticoduodenectomy from 2016 to 2017 were used. Temporal validation was performed using the data of the 452 patients who underwent pancreaticoduodenectomy in 2018. *AI* artificial intelligence, *CR-POPF* clinically relevant postoperative pancreatic fistula, *POPF* postoperative pancreatic fistula, *DL* deep learning, *ML* machine learning.

In the study cohort of 1333 patients, the mean age was 63.4 years, mean BMI was 23.7 kg/m^2^, and 59.8% were men. The most common surgical indication was pancreatic ductal adenocarcinoma (PDAC; n = 531, 39.8%) followed by distal bile duct cancer (n = 291, 21.8%), ampullary cancer (n = 200, 15.0%), duodenal cancer (n = 53, 4.0%), and borderline malignant disease (n = 193, 14.5%), and other benign disease (n = 65, 4.8%). The mean pancreatic duct size measured preoperatively was 3.8 mm, and 63.3% of the patients were classified into soft pancreas intraoperatively. The mean operative time was 330.9 min (Table 1, Fig. 1). The characteristics of participants in the training (n = 881) and test (n = 452) datasets are presented in Supplementary Table 1.

**Table 1:** Characteristics of the study population.

Variable	N = 1333
Age (years)	63.4 ± 10.6
Sex (male)	797 (59.8)
Body mass index (kg/m^2^)	23.7 ± 3.3
Underlying disease (hypertension/diabetes mellitus/cerebrovascular accident)	399 (29.9)/272 (20.4)/30 (2.3)
Laboratory data	
White blood cell (10^3^/μL)	6220 ± 1964
Hemoglobin (g/dL)	12.3 ± 4.9
Total bilirubin (µmol/L)	1.3 ± 2.2
Albumin (g/L)	3.6 ± 6.2
Glucose (mg/dL)	138.2 ± 64.1
Creatinine (mg/dL)	0.7 ± 0.4
Operative indication	
Pancreatic ductal adenocarcinoma	531 (39.8)
Other malignancies	544 (40.8)
Low-grade malignancies	193 (14.5)
Benign disease	65 (4.8)
Neoadjuvant chemotherapy before surgery	88 (6.6)
Pancreatic duct size (mm)	3.8 ± 2.0
Pancreatic texture (soft/hard/firm/unknown)	844 (63.3)/207 (15.5)/267 (20.0)/15 (1.1)
Pylorus preservation	862 (64.7)
Concurrent vessel resection	228 (17.1)
Operative time (min)	330.9 ± 88.2
Intraoperative transfusion	239 (17.9)
Postoperative pancreatic fistula (biochemical leakage/grade B/grade C)	463 (34.7)/83 (6.2)/3 (0.2)
Other postoperative complications	149 (11.2)

Unless otherwise indicated, data presented are the means, with standard deviation in parentheses.

### Associated clinical factors for POPF

POPF and CR-POPF were diagnosed in 555 (41.6%) and 86 (6.4%) participants, respectively. All POPF occurred in 421 (47.8%) and 134 (31.8%) participants, whereas CR-POPF occurred in 59 (6.7%) and 27 (6.0%) participants, in the training and test datasets, respectively. In the multivariable analysis, all preoperative and perioperative clinical factors were included. CR-POPF participants more frequently presented non-PDAC etiology (HR 2.025, 95% CI 1.165–3.519, *p* = 0.012), smaller pancreatic duct size (HR 0.841, 95% CI 0.721–0.980, *p* = 0.027), male sex (HR 1.806, 95% CI 1.103–2.957, *p* = 0.019; Supplementary Table 2) than those without CR-POPF. The results of univariate analyses were shown in Supplementary Tables 3 and 4.

### Body composition factors for POPF

The univariate analyses of the association between the body composition characteristics and the occurrence of POPF and CR-POPF are shown in Supplementary Tables 5 and 6, respectively. Participants with POPF showed higher visceral adipose tissue index (VATI, 43.2 vs. 37.8) and subcutaneous adipose tissue index (SATI, 52.1 vs. 48.1) and higher skeletal muscle index (SMI; 48.4 vs. 46.2) than those without POPF. Myosteatosis presented more frequently in patients without POPF (24.8% vs. 19.6%), and similar trends were observed for CR-POPF patients (higher VATI: 48.3 vs. 39.5, higher SATI: 53.5 vs. 49.5, and higher SMI: 48.1 vs. 47.1) than those without CR-POPF. Patients without CR-POPF had more frequent myosteatosis than those with CR-POPF (22.8% vs. 20.9%).

### Preoperative prediction model for POPF and CR-POPF

**Figure 2:**
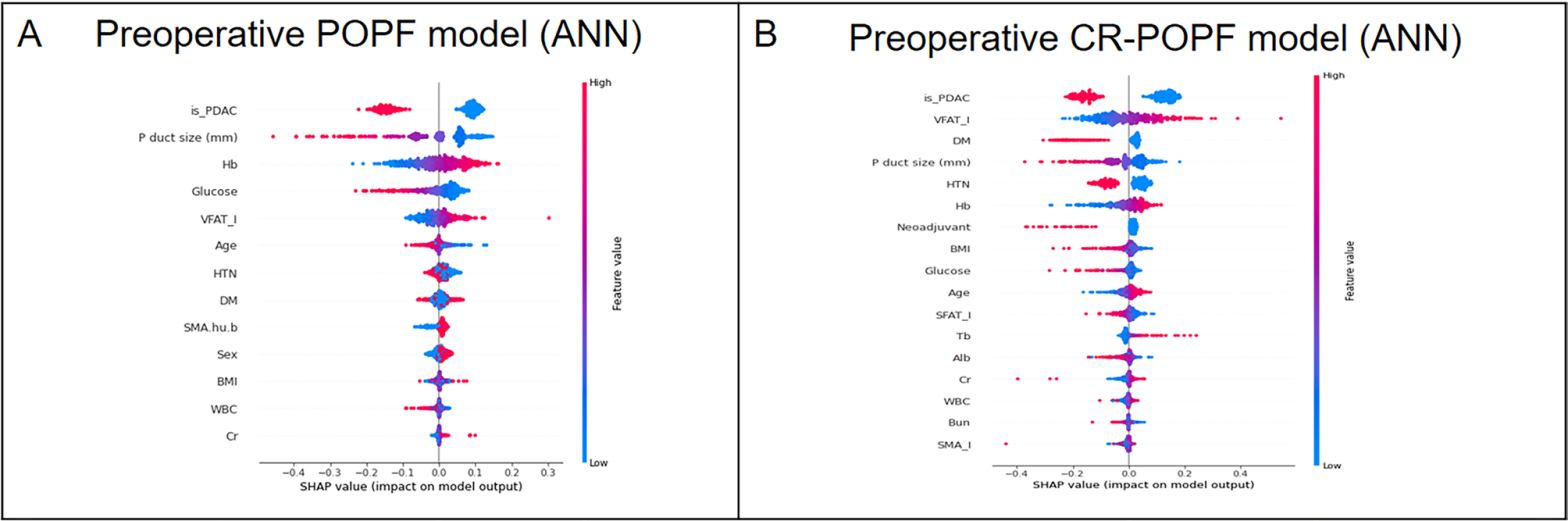
SHAP summary plots of relative feature importance of the selected machine learning models. (**A**) preoperative postoperative pancreatic fistula model, (**B**) preoperative clinically relevant postoperative pancreatic fistula model. Plots order the features based on their importance. Each plot is made up of individual points from the training dataset with a high value being redder and a low value being bluer. *POPF* postoperative pancreatic fistula, *PDAC* pancreatic ductal adenocarcinoma, *Hb* hemoglobin, *VFAT* visceral adipose tissue index, *HTN* hypertension, *DM* diabetes mellitus, *SMA.hu.b* intramuscular adipose tissue index, *BMI* body mass index, *WBC* white blood cell, *Cr* creatinine, *SHAP* sharply additive explanations, *CR-POPF* clinically relevant postoperative pancreatic fistula, *SFAT_I* subcutaneous adipose tissue index.

**Figure 3:**
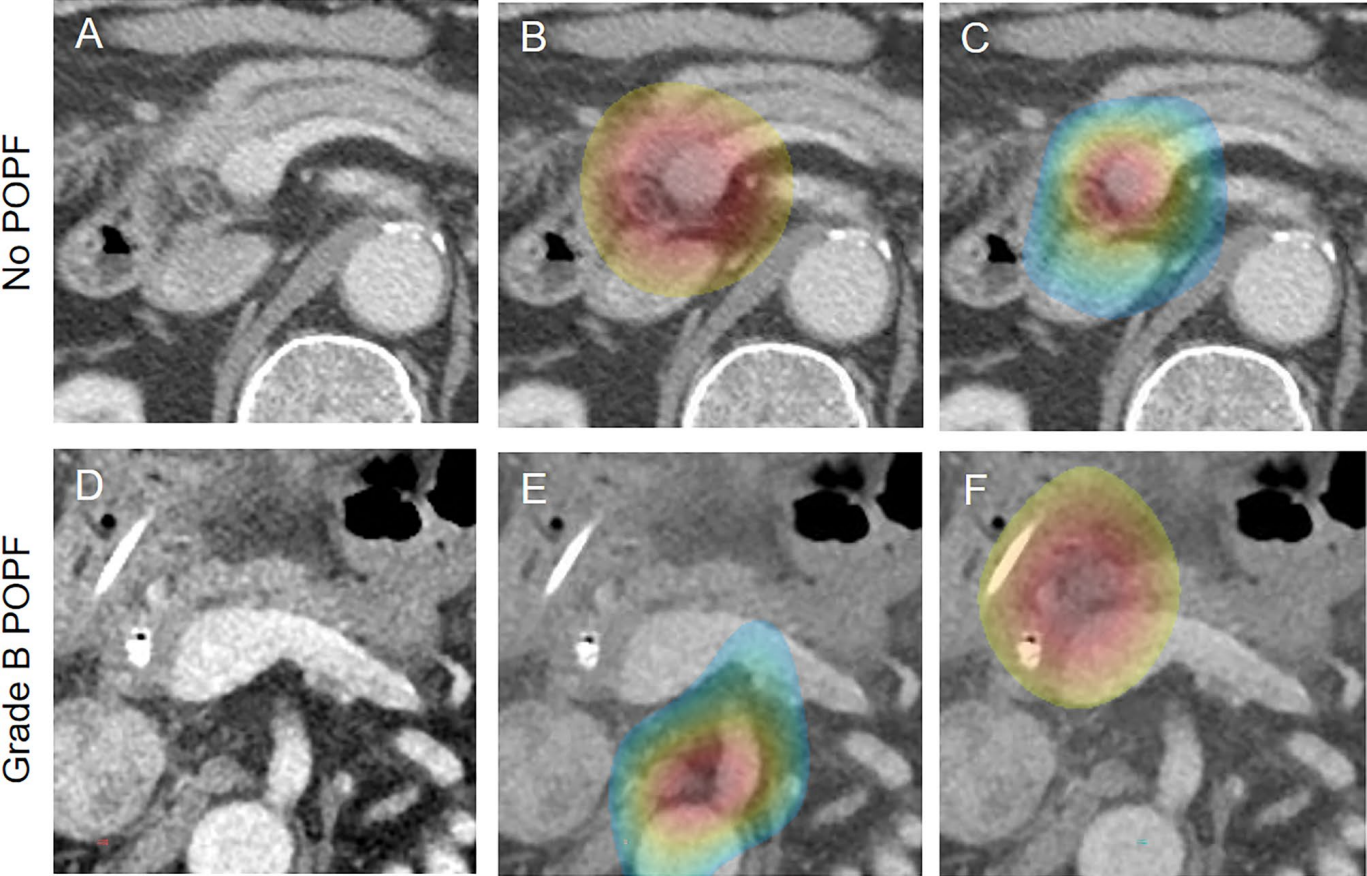
Deep learning attention maps overlaid on computed tomography images of patients in the test dataset. (**A**) A preoperative axial computed tomography image of a 76-year-old man with a pancreatic cancer in the head of pancreas (not shown). In both attention maps of the deep learning models for predicting the occurrence of (**B**) in all postoperative pancreatic fistula and (**C**) clinically relevant postoperative pancreatic fistula, the activated gradient regions focused on the area in the head of pancreas and peripancreatic area around the potential pancreatic resection site. In this patient, the postoperative course was uneventful. (**D**) A preoperative axial computed tomography image of a 74-year-old man diagnosed with distal bile duct cancer (not shown) who experienced Grade B postoperative pancreatic fistula following pancreaticoduodenectomy. In the attention maps of the deep learning models for predicting the occurrence of (**E**) all postoperative pancreatic fistula and (**F**) clinically relevant postoperative pancreatic fistula, the attention of both models is found predominantly in the area around the expected pancreatic resection site. *POPF* postoperative pancreatic fistula, *CR-POPF* clinically relevant postoperative pancreatic fistula.

In the five ML models established using preoperative clinical data, the top commonly selected factors such as non-PDAC etiology, small pancreatic duct size, low glucose level, high hemoglobin, and high VATI for POPF occurrence (Fig. 2). Preoperative CT-based four DL models were developed, and gradient-guided class attention maps showed the areas that the models focused on (Fig. 3). The finally selected model was the soft voting-based ensemble model composed of two ML models (ANN and logistic regression) and one DL model (Inception Net). AUCs of the ensemble model in the training, validation, and test datasets were 0.969, 0.779, and 0.750, respectively. Sensitivity, and specificity were described in Supplementary Table 7. The Roberts model was not included in the ensemble model. The predictive performance of the ensemble model was enhanced as compared to individual ML and DL models.

In the preoperative CR-POPF model, ML models frequently selected non-PDAC etiology, high VATI, absence of diabetes, and smaller pancreatic duct size as important factors predicting CR-POPF. The selected hard voting-based ensemble model comprised three ML models (ANN, TabNet, and random forest) and two DL models (ResNet and ResNeXt); the Roberts model was not included. AUCs of Ensemble model in the training, validation, and test datasets were 0.936, 0.915, and 0.682, respectively, and the ensemble model showed better predictive performance than individual ML and DL models (Table 2).

**Table 2:** Area under the curve values of prediction models for postoperative pancreatic fistula.

	Prediction models	Training set	Validation set	Test set
For postoperative pancreatic fistula	Roberts model	0.662	0.731	0.637
	ML model	0.744	0.769	0.730
	DL model	0.859	0.745	0.714
	Ensemble model	0.969	0.779	0.750
For clinically relevant postoperative pancreatic fistula	Roberts model	0.647	0.623	0.635
	ML model	0.710	0.785	0.623
	DL model	0.978	0.717	0.622
	Ensemble model	0.936	0.915	0.682

For the ML and DL models, values of the single model which showed the best predictive performance are shown.

*ML* clinical and body composition data-based machine learning model, *DL* computed tomography-based deep learning model.

### Comparison between the conventional and the developed models

**Figure 4:**
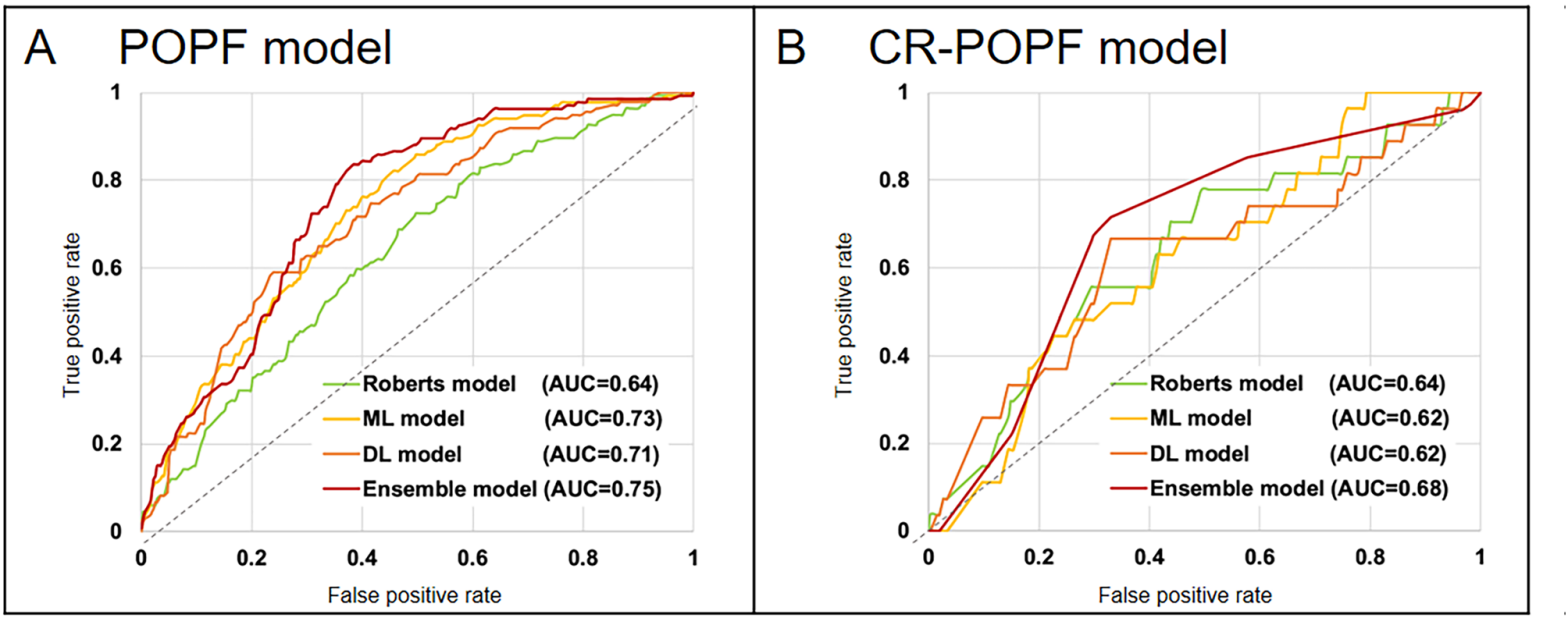
Predictive performance of artificial intelligence models and conventional prediction models for postoperative pancreatic fistula in the test dataset. Receiver operating characteristic curves of the ensemble models (red), machine learning models (yellow), deep learning models (orange), and prior models (Roberts model [green]) are plotted. The ensemble models showed the best predictive performance for preoperatively predicting all postoperative pancreatic fistula (**A**) and clinically relevant postoperative pancreatic fistula (**B**). *POPF* postoperative pancreatic fistula, *CR-POPF* clinically relevant postoperative pancreatic fistula, *DL* deep learning, *ML* machine learning.

The predictive performance of the Roberts model and the preoperative ensemble model were compared to preoperatively predict POPF, and the preoperative ensemble model showed better performance (AUC, 0.750 vs. 0.637; *p* < 0.001); however, comparable predictive performance was observed between the preoperative ensemble and Roberts models for CR-POPF prediction (AUC, 0.682 vs. 0.635; *p* = 0.42). (Table 2, Fig. 4).

### Changing AUC pattern according to the CR-POPF incidence

**Figure 5:**
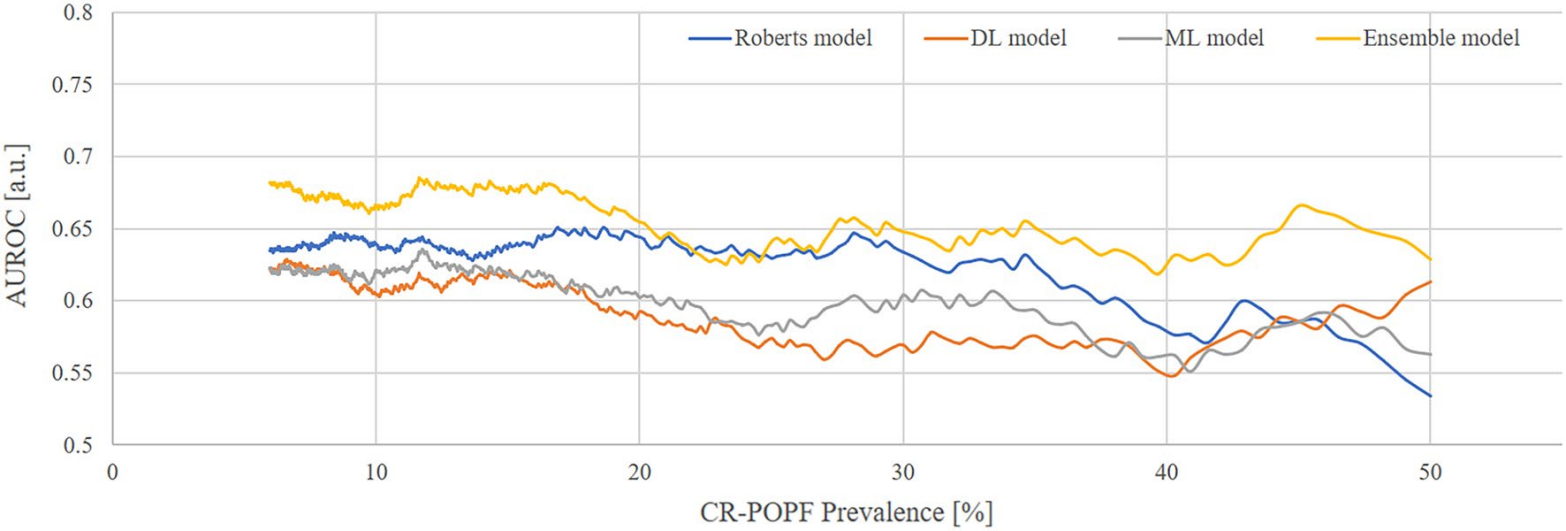
Changing pattern of area under the curve value according to the clinically relevant pancreatic fistula incidence. The value of area under the curve of the ensemble models (yellow), machine learning models (gray), deep learning model (orange), and conventional models (Roberts model [blue]) for clinically relevant pancreatic fistula are plotted. Preoperative ensemble model for clinically relevant pancreatic fistula showed AUC ≥ 0.7 after 18% of the clinically relevant pancreatic fistula rate; AUCs of the ensemble model showed better performance than other models.

The low ratio of CR-POPF could affect model development because of the potential bias toward major cases and the negative impact of the model’s ability to learn. In this study, the CR-POPF incidence was relatively lower than that in other institutions, and we investigated changing pattern of model performance when the ratio of control and event were adjusted from 6.5% to 50%. The preoperative ensemble model for CR-POPF showed optimal performance AUCs regardless of the incidence ratio of CR-POPF, whereas the AUC of the Robert models decreased to approximately 30% of CR-POPF (Fig. 5).

### Postoperative prediction models and the alternative fistula risk score

An all-inclusive prediction model was developed using preoperative, intraoperative, and postoperative variables. To predict the POPF, ML models selected non-PDAC etiology, soft pancreatic texture, high drain amylase level at postoperative day 1, and the absence of vascular resection as the top features. The AUCs of the ensemble model in the training, validation, and test dataset were 0.936, 0.832, and 0.787, respectively. The ensemble model showed higher AUCs than the alternative fistula risk score (a-FRS)^3^ in predicting the POPF (0.787 vs. 0.696; *p* < 0.001). There was no difference in CR-POPF prediction accuracy between the comprehensive ensemble and a-FRS models (0.685 vs. 0.667; *p* = 0.59; Supplementary Table 8).

## Discussion

In this study, we developed AI-based models for predicting all POPF and CR-POPF in a large sample of 1333 patients undergoing PD. The preoperative ensemble model for POPF outperformed the prediction value compared to the conventional, ML, and DL models. The preoperative ensemble model for CR-POPF showed comparable performance, but had better predictive performance than the Roberts model after adjustment of CR-POPF incidence. The postoperative ensemble model for POPF showed better prediction value compared to the a-FRS model.

Previous studies reported that 10–40% of the patients who undergo PD experience CR-POPF^2^. Many POPF prediction models were published that included common factors such as small pancreatic duct, soft pancreatic texture, and high BMI^9^, and are commonly used at the bedside because they are simple, comprise only two or three variables, and showed good performance.

However, recent studies have reported novel risk factors for POPF. Pathologic studies showed that fatty pancreas are associated with POPF, whereas atrophied and fibrotic pancreas have a protective role^4,8^, and the recent improvement of CT technology identified novel potential factors for predicting POPF: Shi et al. showed that a higher pancreatic parenchymal-to-portal venous iodine concentration ratio measured on dual-energy CT was associated with less histologic fibrosis and greater risk of POPF^14^. Moreover, anthropomorphic studies showed that CT-based body composition data may help predict postoperative complications, such as POPF and poor survival^15–20^. Prior studies^16,17^ have consistently suggested the impact of high visceral obesity on POPF incidence, whereas the impact of skeletal muscle mass on POPF incidence remains controversial: several studies^17,18^ have shown a protective effect whereas others^15,19,20^ have failed to identify such effect. Studies reporting the impact of myosteatosis are limited, and a study with 139 participants^15^ showed that patients with lower SMD more frequently developed CR-POPF than those with higher SMD. In our study, high VATI was associated with both all POPF and CR-POPF incidence, which matches the results of prior studies.

The diversification of the pancreatic surgery environment has increasingly necessitated the development of a comprehensive prediction model that includes various factors. A recent meta-analysis revealed other POPF risk factors, including male sex, blood transfusion, vascular resection, and neoadjuvant chemotherapy^9^. In this study, non-PDAC etiology, small pancreatic duct size, low glucose level, high hemoglobin, and high VATI were risk factors with POPF occurrence in representative ML models. The pancreatic parenchyma in patients with non-PDAC etiology is characterized by soft and abundant tissue. Typically, this includes non-dilated pancreatic ducts, which serve as iconic risk factors. Additionally, individuals with high VATI, indicative of high visceral obesity, are also recognized as a risk factor. Most of risk factors align with findings from previous studies^9,18^. There might be two confusing factors. High hemoglobin levels do not seem to be a standalone risk factor. It could be associated with male sex and high visceral fat, both of which are known risk factors^4,9^. Low preoperative glucose levels may be related with soft and fatty pancreas, as shown in previous meta-analysis^9^. The important feature of ML and DL models are integration of big data, and AI is a suitable tool for this research task. Several studies have evaluated ML and DL prediction models. Kambakamba et al. reported an ML model developed using data of texture analysis from 110 patients who were matched with POPF and non-POPF groups of 55 and 55 patients, respectively, and showed that ML-based texture analysis could predict fibrotic change of pancreatic parenchyma (AUC; 0.84) and POPF (AUC; 0.95)^21^. The authors adjusted the control group sample for efficient training of the AI model; however, in real-world practice, the incidence of CR-POPF is lower than in the experimental setting. Han et al. reported an ML model using 38 clinical variables from 1769 patients, and the CR-POPF incidence was 12.5% and the AUC of the ML model was 0.74^22^. Shen et al. reported various ML models using clinical and radiomics data from 2421 patients, and the CR-POPF rate was 12.5% and ML model had an AUC of 0.83^23^. Mu et al. developed a DL model using CT-based data from 583 patients that showed a CR-POPF rate of 13.6% (AUC 0.85, with better performance compared to FRS)^13^. Recently, ML-based models using preoperative factors have been reported. Ganjouei et al. developed an ML model (AUC 0.72) based on clinical factors which was useful for quick use. They selected the XGboost model among several ML models; however, a comparison with prior models was not performed^24^. Other studies reported ML models based on preoperative clinical factors and radiologic data from CT scans. However, they included a small number of the patients which led to the potential of overfitting, and radiologic data were processed manually^25,26^. In this study, ML, DL, and ensemble models were applied for each purpose. As in previous studies, ML models were suitable for modeling a collection of various clinical and body composition data. To develop DL models, raw CT data were used, without pancreatic segmentation or complex manual feature-selection processes, whereas previous studies extracted radiomic data for texture analysis in a labor-intensive task that requires large human resources^21,27^. Preoperative and comprehensive prediction models are provided for suitable use of various clinical settings. Moreover, we provided POPF and CR-POPF models separately because the CR-POPF rate was 6.4%, which indicates lower incidence compared to the published data. Class imbalance could have affected the model’s learning capacity, and several solutions were introduced such as semi-supervised learning, data augmentation, resampling, and ensemble modeling^28–30^. In this study, an ensemble method after individual ML modeling was used. Furthermore, we adjusted the ratio of CR-POPF and found that performance of preoperative ensemble model for CR-POPF was stable when the ratio of CR-POPF increased to less than 20%, and the model consistently outperformed the Roberts model, except in the one with 20–25% CR-POPF incidence. The Roberts model showed decreased performance with high CR-POPF incidence (> 30%), indicating that BMI and pancreatic duct size were insufficient risk factors in high CR-POPF incidence. In contrast, the ensemble model demonstrated a consistent performance across diverse CR-POPF rates owing to its incorporation of various risk factors during the modeling process. However, our comprehensive model showed similar predictive performance compared to the conventional a-FRS model. The comprehensive CR-POPF model comprised logistic regression, ResNeXt, and a-FRS model. A crucial portion of the comprehensive CR-POPF model may have already been occupied in the a-FRS model that included pancreatic texture, pancreatic duct size, and BMI, which are well-known CR-POPF risk factors. The additional logistic regression ML model included risk factors such as high amylase in drainage on POD1, high VATI, absence of diabetes; however, these factors in the ML model did not provide incremental value for the final model. Therefore, predictive performances of the ensemble and a-FRS models were comparable.

There are several limitations of this study. The decision to utilize three years of input data was driven by the availability of well-structured input data, an adequate number of patients for model establishment, and a recent decrease in the incidence of CR-POPF. The model development and validation processes were performed using data from a single center. There may be discrepancies in the postoperative management because multiple surgeons participated with this study. However, we standardized the critical pathway after surgery and unified the surgical procedures to minimize discrepancies. Stringent internal validation was performed because data for external validation were unavailable. The CR-POPF incidence is relatively lower than in other centers, which may be related with the unified procedures based on cumulative experiences and high volume of surgeries^31^. However, it may hinder determination of the statistical significance of several factors. However, we minimized this shortcoming by performing temporal validation. Despite the abundance of samples and the use of an ensemble model, the potential risk of overfitting may be a limitation during segmentation into multiple datasets and utilization of ML models.

A preoperative ensemble model for POPF provide better predictive performance than conventional model in preoperative clinical settings. Furthermore, developed ensemble model showed stable performance for predicting postoperative pancreatic fistula compared to prior model nevertheless of incidence of CR-POPF. This preoperative model could be useful for identifying risky patients in clinical studies for pancreatectomy and could help clinicians decide the immediate postoperative management in any suspicious situation.

## Methods

### Study population

This study was reported in line with the STROBE, and STROCSS^32^ criteria. The Institutional Review Board of Asan Medical Center approved the experimental protocol of this retrospective study and waived the need for informed consent (IRB No: 2021-0559). All methods were performed in accordance with good clinical practice guidelines and adhered to the principles outlined in the Declaration of Helsinki. The study was registered at cris.nih.go.kr (KCT0008156). Patients who underwent pancreaticoduodenectomy (PD) for periampullary diseases from 2016 to 2018 were enrolled. Exclusion criteria included: (a) incomplete details according to risk scores for POPF (Roberts model^7^); (b) absence of contrast-enhanced CT images during the 30 days before surgery; and (c) suboptimal CT quality due to severe artifact. Among the 1333 participants, data of those who underwent surgery from 2016 to 2017 (881 patients) and in 2018 (452 patients) were used as the training dataset and model validation, respectively (Fig. 1).

### Study endpoints

The primary endpoints were the occurrence of all POPF and CR-POPF. POPF was defined according to the International Study Group in Pancreatic Surgery definition^33^, and grades B and C POPF were classified as CR-POPF.

### Data collection

Data on patients’ demographics, pre- and perioperative clinical data, preoperative CT images, intraoperative findings, and pathologic diagnosis were collected. Various CT scanners and image acquisition techniques were used. Details of CT acquisition were provided in the Supplementary Method and Supplementary Table 9; portal venous phase (PVP) CT images were used in the analysis. For body composition assessment, a single axial CT image at the level of lower endplate of the 3rd lumbar vertebra was used^34,35^ to measure the cross-sectional areas of total abdominal wall muscle, subcutaneous adipose tissue, and visceral adipose tissue with artificial intelligence software (AID-UTM, iAID inc, Seoul, Republic of Korea)^36^. The body composition parameters were normalized by division by the height squared (cm^2^/m^2^) and then reported as indices, including SMI, SATI, and VATI. Skeletal muscle density (SMD), which represents the degree of myosteatosis, was quantified as the mean HU of the skeletal muscle area (cutoff: 41 and 33 HU for non-overweight and overweight patients, respectively)^37^. Details of body composition analysis are provided in Supplementary Method.

### Surgical techniques and postoperative care

All surgical procedures were performed by experienced pancreatic surgeons using described operative procedures. Briefly, the pancreas was divided at the left side of the superior mesenteric vein, and pancreatic texture and pancreatic duct size were assessed intraoperatively by the attending surgeon. After a roux limb formation, end-to-side pancreaticojejunostomy (PJ) was performed. Non-absorbable monofilament was used for out-layer anastomosis with interrupted or continuous sutures. All surgeons performed duct-to-mucosa PJ anastomosis with an internal plastic stent, which was selected according to the size of the pancreatic duct. At surgery completion, two or three drains were placed adjacent to the PJ anastomosis and on the right side of the superior mesenteric arterial resection margin.

Postoperatively, serum and peripancreatic drain fluid amylase levels were routinely measured on postoperative days 1, 3, and 5; a contrast-enhanced CT scan was performed on days 5 to detect any complications. The peripancreatic drains were removed if there was no evidence of leakage on postoperative days 3–5. In cases with biochemical leakage, no additional treatment was performed. In case of leakage or suspicion of infective complications, the peripancreatic drains were left in situ, and antibiotics were administered at the discretion of the attending physician. Percutaneous or endoscopic drainage was performed according to the location of fluid collection. Reoperation was performed in patients with uncontrolled infection or unstable vital signs despite proper drainage and antibiotic use.

### Model development for POPF prediction

A schematic representation of the developed models is provided in Fig. 1, and details of model development are provided in Supplementary Method. Using the training dataset, we developed models for predicting POPF and CR-POPF using preoperative data. We developed five ML models (artificial neural network [ANN], tabular network, logistic regression, random forest, and gradient boosting) utilizing the clinical information and body composition data. To train the DL models, the training dataset was divided into the training and validation subsets, and four DL models (ResNet, DenseNet, ResNeXt, and Inception net) were created utilizing preoperative CT data. Ensemble voting was used to combine the developed ML models, DL models, and the prior models (Roberts model^7^) with soft or hard voting, and the model with the highest accuracy in the validation subset was chosen. Finally, the single preoperative comprehensive model was selected, and the predictive performance was evaluated using the separate test dataset. The codes used in this work are available in the GitHub repository (https://github.com/nolife119/POPF_ensemble).

### Statistical analysis

The sample size was calculated based on the area under the receiver operating characteristic curve (AUC). Based on previous studies, we hypothesized that the AUC would be 0.750. The proportion of sample with a POPF was 6–7%. The two-sided significance level (α) was set at 5%, and the statistical power (1-β) was set at 95%. The final number of subjects required for this study was 782. The chi-square test was used to compare categorical data, and the independent *t*-test was used to compare continuous data. Binary logistic regression analyses were used to evaluate the association between the variables and the occurrence of POPF and CR-POPF. The predictive performance of the selected models was assessed from the receiver operating characteristics (ROC) curve analysis, and the area under the ROC curve (AUC) with confidence interval (CI) was calculated. The sensitivity, specificity, and F1 score were obtained with the models’ cutoff value showing the highest accuracy in the validation subset. Analyses were performed using SAS version 9.4 (SAS Institute, Cary, NC, USA). Two-sided *p* < 0.05 were considered statistically significant.

### Supplementary Information

Supplementary Information.

## Data Availability

The codes used in this work are available in the GitHub repository: https://github.com/nolife119/POPF_ensemble.
