# Supplementary material for: Deep learning-based prediction of post-pancreaticoduodenectomy pancreatic fistula
Source: Sci Rep. 2024 Mar 1;14:5089. doi: 10.1038/s41598-024-51777-2 (PMC10907568; doi:10.1038/s41598-024-51777-2)
Supplement: Supplementary file 1 — Supplementary Information. [file 41598_2024_51777_MOESM1_ESM.pdf]

Supplementary contents

Supplementary table 1. Characteristics of the study population

Supplementary table 2. Clinical risk factors for postoperative pancreatic fistula

Supplementary table 3. Characteristics of the patients with POPF

Supplementary table 4. Characteristics of the patients with CR-POPF

Supplementary table 5. Body composition characteristics of patients with and without POPF

Supplementary table 6. Body composition characteristics of patients with and without CR-POPF

Supplementary table 7. Predictive performance of preoperative models in the training, validation, and test set

Supplementary table 8. Area under the curve values of postoperative prediction models for postoperative pancreatic fistula

Supplementary table 9. CT imaging techniques used for the development and test datasets.

Supplementary method

Supplementary table 1. Characteristics of the study population

**Variable**	**Training dataset (n=881)**	**Test dataset (n=452)**	***P*-value**
Age (years)	62.7 ± 10.7	64.8 ± 10.5	0.001
Sex (male)	537 (61.0)	260 (57.5)	0.238
Body mass index (kg/m^2)	23.7± 3.5	23.7 ±3.0	0.849
Hypertension	208 (23.6)	191 (42.3)	<0.001
Diabetes	140 (15.9)	132 (29.2)	<0.001
Cerebrovascular disease	6 (0.7)	24 (0.3)	<0.001
Laboratory data			
White blood cell (10^3/uL)	6265 ±2075	6134 ±1727	0.222
Hemoglobin (g/dL)	12.5± 6.0	12.1± 1.6	0.160
Total bilirubin (µmol/L)	1.5 ±2.4	1.1 ±1.9	0.001
Albumin (g/L)	3.6 ±7.6	3.4 ±0.4	0.518
Glucose (mg/dL)	137.9 ±63.8	139.0 ±64.8	0.754
Blood urea nitrogen (mg/dL)	13.9 ±5.5	14.2 ±5.7	0.308
Creatinine (mg/dL)	0.7± 0.4	0.8± 0.3	0.373
Operative indication			0.280
Pancreatic cancer	344 (39.5)	187 (41.9)	
Other malignancies	354 (40.7)	190 (42.6)	
Low-grade malignancies	139 (16.0)	54 (12.1)	
Benign disease	33 (3.8)	15 (3.4)	
Neoadjuvant chemotherapy	54 (6.1)	34 (7.5)	0.352
Pancreatic texture (soft/hard/firm)	538 (61.1)/153(17.4)/177(20.1)	306 (67.7)/54(11.9)/90(19.9)	0.069
Pancreatic duct size (mm)	3.7 ±2.1	4.0± 1.9	0.003
Concurrent vessel resection	149 (16.9)	79 (17.5)	0.818
Operative time (min)	360.3 ±80.7	273.5 ±72.6	<0.001
Intraoperative transfusion	164 (18.6)	75 (16.6)	0.407
Pylorus preservation	635 (72.6)	227 (50.2)	<0.001
Drain amylase POD3	954.1 ±3618.4	483.3 ±1195.6	<0.001
CR-POPF (B/C)	56 (6.4) / 3 (0.3)	27 (6.0)/0	0.639

Note—Unless otherwise indicated, data that are presented are means, with standard deviation in parentheses.
Abbreviations: POD, postoperative day; CR-POPF, clinically relevant postoperative pancreatic fistula.

Supplementary table 2. Clinical risk factors for postoperative pancreatic fistula

	For POPF			***For CR-POPF***		
**Variables**	**HR**	**95% CI**	***P*-value**	**HR**	**95% CI**	***P*-value**
Non-PDAC etiology	2.464	1.849–3.282	<0.001	2.025	1.165–3.519	0.012
Pancreatic duct size	0.799	0.740–0.863	<0.001	0.841	0.721–0.980	0.027
Male sex				1.806	1.103–2.957	0.019
Glucose level	0.997	0.995–0.999	0.006			
Body mass index	1.040	1.000–1.080	0.047			
Pancreatic texture (Soft[ref] / hard, firm)	0.401	0.3–0.537	<0.001	0.575	0.312–1.059	0.076

Note—Unless otherwise indicated, data presented are means, with standard deviation in parentheses. Abbreviations: POPF, postoperative pancreatic fistula; CR-POPF, clinically relevant postoperative pancreatic fistula; PDAC, pancreatic ductal adenocarcinoma.

Supplementary table 3. Characteristics of the patients with POPF

**Variable**	**No-POPF (n=778)**	**POPF (n=555)**	***P*-value**
Age (years)	64.1 ± 10.1	62.5 ± 11.3	0.007
Sex (male)	455 (58.5)	342 (61.6)	0.257
Body mass index (kg/m^2)	23.4 ± 3.0	24.2 ± 3.7	<0.001
Hypertension	251 (32.3)	148 (26.7)	0.029
Diabetes	193 (24.8)	79 (14.2)	<0.001
Cerebrovascular disease	23 (3.0)	7 (1.3)	0.041
Laboratory data			
WBC (10^3/uL)	6206 ± 2039	6241 ± 1857	0.744
Hemoglobin (g/dL)	12.1 ± 1.7	12.7 ± 7.4	0.027
Total bilirubin (µmol/L)	1.3 ± 2.1	1.4 ± 2.5	0.521
Albumin (g/L)	3.7 ± 8.1	3.4 ± 0.4	0.450
Glucose (mg/dL)	146.9 ± 71.8	126.2 ± 49.0	<0.001
Blood urea nitrogen (mg/dL)	14.0 ± 5.8	14.1 ± 5.3	0.688
Creatinine (mg/dL)	0.7 ± 0.2	0.8 ± 0.6	0.047
Operative indication			
PDAC/non-PDAC	419 (53.9) /359 (46.1)	112 (20.2) / 443 (79.8)	<0.001
Minimally invasive surgery	102 (13.1)	113 (20.4)	<0.001
Neoadjuvant chemotherapy	66 (8.5)	22 (4.0)	0.001
Pancreatic texture (soft/hard/firm/ unknown)	389(50.0)/171(22.0)/208(26.7)/10(1.3)	455 (82.0)/36(6.5)/59(10.6)/5(0.9)	<0.001
Pancreatic duct size (mm)	4.3 ± 2.3	3.1 ± 1.4	<0.001
Concurrent vessel resection	179 (23.0)	49 (8.8)	<0.001
Operative time (min)	328.4 ± 88.4	334.3 ± 87.9	0.232
Intraoperative transfusion	169 (21.7)	70 (12.6)	
Pylorus preservation	481 (62.1)	381 (69.0)	0.010
Drain amylase POD3	136.0 ± 279.1	1708.1 ± 4526.7	<0.001
CRP POD 3	8.1 ± 4.7	8.9 ± 5.4	0.014
Postoperative hospital stay	12.2 ± 6.2	13.9 ± 8.3	<0.001
Other complications	63 (8.1)	86 (15.5)	<0.001

Note—Unless otherwise indicated, data that are presented are means, with standard deviation in parentheses.

Abbreviations: PDAC, pancreatic ductal adenocarcinoma; POD, postoperative day; CRP, C-reactive protein

Supplementary table 4. Characteristics of the patients with CR-POPF

**Variable**	**No CR-POPF (n=1247)**	**CR-POPF (n=86)**	***P*-value**
Age (years)	63.4 ± 10.7	64.2 ± 10.6	0.482
Sex (male)	733 (59.0)	62 (72.1)	0.017
Body mass index (kg/m^2)	23.7 ± 3.3	24.4 ± 3.2	0.063
Hypertension	368 (29.6)	29 (33.7)	0.465
Diabetes	260 (20.9)	12 (14.0)	0.130
Cerebrovascular disease	29 (2.3)	1 (1.2)	0.717
Laboratory data			
WBC (10^3/uL)	6.2 ± 1.9	6.6 ± 2.2	0.084
Hemoglobin (g/dL)	12.3 ± 5.1	12.5 ± 2.1	0.761
Total bilirubin (µmol/L)	1.3 ± 2.2	2.0 ± 3.3	0.054
Albumin (g/L)	3.6 ± 6.4	3.3 ± 0.4	0.735
Glucose (mg/dL)	139.3 ± 65.6	123.1 ± 33.1	0.001
Blood urea nitrogen (mg/dL)	13.9 ± 5.4	15.2 ± 7.4	0.131
Creatinine (mg/dL)	0.7 ± 0.4	0.9 ± 0.6	0.032
Operative indication			
PDAC/non-PDAC	510 (41.1)/ 732 (58.9)	18 (20.9)/68(79.1)	
Minimally invasive surgery	205 (16.5)	10 (11.6)	0.289
Neoadjuvant chemotherapy	84 (6.8)	3 (3.5)	0.363
Pancreatic texture (soft/hard/firm/unknown)	769(61.9)/200(16.1)/259(20.9)/14(1.1)	70(81.4)/7(8.1)/8(9.3)/1(1.2)	0.001
Pancreatic duct size (mm)	3.9 ± 2.1	3.1 ± 1.5	0.001
Concurrent vessel resection	221 (17.8)	6 (7.0)	0.007
Operative time (min)	330.5 ± 87.6	335.6 ± 97.5	0.604
Intraoperative transfusion	222 (17.9)	16 (18.6)	0.884
Pylorus preservation	806 (65.2)	52 (60.5)	0.414
Drain amylase POD3	653.8 ± 2345.3	2842.2 ± 7718.6	0.001
CRP POD 3	8.3 ± 4.9	11.1 ± 6.3	0.001
Postoperative hospital stay	12.0 ± 5.6	26.2 ± 12.4	0.001
Other complications	99 (8.0)	50 (58.1)	0.001

Note—Unless otherwise indicated, data that are presented are means, with standard deviation in parentheses.

Abbreviations: PDAC, pancreatic ductal adenocarcinoma; POD, postoperative day; CRP, C-reactive protein

Supplementary table 5. Body composition characteristics of patients with and without POPF

**Variable**	**No-POPF (n=778)**	**POPF (n=555)**	***P*-value**
VATI	37.8 ± 21.2	43.2 ± 26.3	<0.001
SATI	48.1 ± 24.7	52.1 ± 27.6	0.005
SMI	46.2 ± 7.8	48.4 ± 15.9	<0.001
Myosteatosis	193 (24.8)	109 (19.6)	0.028

Note—Unless otherwise indicated, data that are presented are means ± standard deviation or number with percentage in parentheses.
Abbreviations: SATI, subcutaneous adipose tissue index; SMI, skeletal muscle index; VATI, visceral adipose tissue index

Supplementary table 6. Body composition characteristics of patients with and without CR-POPF

**Variable**	**No CR-POPF (n=1247)**	**CR-POPF (n=86)**	***P*-value**
VATI	39.5 ± 23.7	48.3 ± 20.6	0.001
SATI	49.5 ± 25.9	53.5 ± 28.3	0.164
SMI	47.1 ± 12.1	48.1 ± 7.6	0.444
Myosteatosis	284 (22.8)	18 (20.9)	0.79

Note—Unless otherwise indicated, data that are presented are means ± standard deviation or number with percentage in parentheses.
Abbreviations: SATI, subcutaneous adipose tissue index; SMI, skeletal muscle index; VATI, visceral adipose tissue index

Supplementary table 7. Predictive performance of preoperative models in the training, validation and test set

			AUROC	Sensitivity	Specificity	F1 score
Training set	For POPF	Roberts model	0.662	0.797	0.451	0.666
		ML model	0.744	0.725	0.625	0.680
		DL model	0.859	0.848	0.710	0.784
		Ensemble model	0.969	0.928	0.818	0.873
	For CR-POPF	Roberts model	0.647	0.845	0.348	0.180
		ML model	0.710	0.707	0.560	0.208
		DL model	0.978	1.000	0.925	0.699
		Ensemble model	0.936	0.966	0.675	0.337
Validation set	For POPF	Roberts model	0.731	0.806	0.543	0.695
		ML model	0.769	0.722	0.691	0.698
		DL model	0.745	0.778	0.605	0.700
		Ensemble model	0.779	0.806	0.654	0.734
	For CR-POPF	Roberts model	0.623	0.857	0.390	0.118
		ML model	0.785	0.714	0.596	0.141
		DL model	0.717	0.286	0.856	0.133
		Ensemble model	0.915	1.000	0.616	0.200
Test set	For POPF	Roberts model	0.637	0.716	0.509	0.497
		ML model	0.730	0.709	0.629	0.548
		DL model	0.714	0.799	0.519	0.543
		Ensemble model	0.750	0.828	0.619	0.607
	For CR-POPF	Roberts model	0.635	0.778	0.456	0.151
		ML model	0.623	0.556	0.619	0.147
		DL model	0.622	0.259	0.866	0.154
		Ensemble model	0.682	0.704	0.692	0.215

Note—Unless otherwise indicated, data that are presented are means, with standard deviation in parentheses. For the ML and the DL models, values of the single model which showed the best predictive performance are shown.

Abbreviations: AUROC, area under the receiver operating characteristic curve; POPF, postoperative pancreatic fistula; CR-POPF, clinically relevant postoperative pancreatic fistula; ML, clinical and body composition data-based machine learning model; DL, computed tomography-based deep learning model; FRS, fistula risk score.

Supplementary table 8. Area under the curve values of postoperative prediction models for postoperative pancreatic fistula

		Training set	Validation set	Test set
For POPF	Alternative fistula risk score model	0.721	0.795	0.696
	ML model	0.810	0.801	0.773
	DL model	0.859	0.832	0.714
	Ensemble model	0.936	0.795	0.787
For CR-POPF	Alternative fistula risk score model	0.666	0.624	0.667
	ML model	0.616	0.689	0.636
	DL model	-	-	0.622
	Ensemble model	0.834	0.799	0.685

Note—Unless otherwise indicated, data presented are means, with standard deviation in parentheses. For the ML and DL models, values of the single model which showed the best predictive performance are shown.

Abbreviations: ML, clinical and body composition data-based machine learning model; DL, computed tomography-based deep learning model; POPF, postoperative pancreatic fistula; CR-POPF, clinically relevant postoperative pancreatic fistula;

Supplementary table 9. CT imaging techniques used for the development and test datasets

CT techniques	Training dataset (n = 881)	Test dataset (n = 452)
**Configuration**		
16–32 detectors	183 (20.8)	69 (15.3)
64 detectors	697 (79.1)	381 (84.3)
≥128 detectors	1 (0.1)	2 (0.4)
**Tube voltage, kVp**		
90–110	169 (19.2)	275 (60.8)
120	699 (79.3)	174 (38.5)
130–140	13 (1.5)	3 (0.7)
**Slice thickness, mm**		
≤2.5	244 (27.7)	125 (27.7)
3	503 (57.1)	276 (61.2)
3.75–5	132 (15.0)	50 (11.1)
7	2 (0.2)	0 (0.0)
**CT vendors**		
Siemens	565 (64.1)	301 (66.7)
GE	277 (31.4)	137 (30.4)
Toshiba	19 (2.2)	8 (1.8)
Philips	16 (1.8)	4 (0.9)
Hitachi	4 (0.5)	1 (0.2)
**Pixel size, mm**		
0.53–0.7	588 (66.7)	318 (70.4)
0.7–0.83	293 (33.3)	134 (29.6)

Data are presented as n (%).
Abbreviation: kVp, peak kilovoltage.

Supplementary method

CT techniques

All CT images were obtained using 16-detector or higher CT systems. For contrast enhancement, the total volume of non-ionic iodinated contrast medium was stratified according to the patient's body weight (approximate rate, 2 mL/kg; maximum rate,150 mL), and an automatic power injector was used to deliver the contrast agent intravenously (3 mL/s). Portal venous phase (PVP) images were obtained at 70–75 seconds after contrast injection. Images were reconstructed using filtered back projection (B30f, B30s, B41f, B41s) or iterative reconstruction (I30s, I30f). Pixel size ranged from 0.53 to 1.11 mm.

Body composition analysis

A single PVP axial CT image at the level of the lower endplate of the 3rd lumbar vertebra was used.[1,2] The cross-sectional areas of the total abdominal wall muscle (skeletal muscle area; including psoas, paraspinal, transversus abdominis, rectus abdominis, quadratus lumborum, and internal and external obliques), subcutaneous adipose tissue, and visceral adipose tissue were measured with pre-established thresholds (from −29 to +150 Hounsfield unit [HU] for skeletal muscle area and from −190 to −30 HU for subcutaneous and visceral adipose tissues).[3] The body composition parameters were normalized by being divided to the patient height squared (cm^2/m^2) and reported as indices, including the visceral adipose tissue index (VATI), subcutaneous adipose tissue index (SATI), and skeletal muscle index (SMI). Skeletal muscle density (SMD), which represents the degree of myosteatosis, was quantified as the mean HU of the skeletal muscle area; the cutoff points for the presence of myosteatosis were set at 41 and 33 HU for non-overweight and overweight patients, respectively.[4]

Model development

Machine learning models

The clinical information and body composition data extracted from the training dataset was used to develop machine learning models. In cases of missing values, the median value of each variable was imputed. Five machine learning models (artificial neural network [ANN], tabular network [TabNet], logistic regression, random forest, and gradient boosting) were employed.[5-7] ANN was trained by an Adam optimizer with a batch size of 257 and a learning rate of 4e-3. The training part of TabNet was performed using the Adam optimizer (learning rate, 0.1; batch size, 64). The binary cross-entropy loss function (i.e., average difference between the predicted and actual probabilities) was used for the ANN and TabNet. Linear LR with L2-regularization and Kernel SVM with Gaussian kernel were used. The number of trees in RF and GB was 100. All machine learning models, except for ANN and TabNet, were trained using the Scikit-learn library on Python 3.8.[6] The deep learning library Keras 2.5 version was used for the development of the ANN and TabNet models.[7]

Deep learning models

We developed two-dimensional (2D) convolutional neural network-based deep learning models. CT images underwent several pre-processing steps, including resampling, intensity normalization, augmentation, and cropping. All CT images were resampled to pixels of 0.5×0.5 mm^2 using spline interpolation to decrease the variability between scans.[8,9] The image intensities were normalized from 0 to 1 by using the limit of lower and upper HU as −200 and 300, respectively. Image augmentation techniques, such as rotation, shearing, scaling, and modification of the image brightness, were applied to enhance the size of the training dataset.[10] For data augmentation, we used rotation angles ranging from −5° to 5°, with an interval of 1° and shifting of brightness ranging from −0.1 to 0.1 (interval, 0.01). Moreover, scaling and shearing ratios of heights and widths ranging from 95% to 105% with an interval of 1% were utilized. Each data augmentation technique was applied on a 50-50 chance, and the parameters were randomly selected within a predefined range. As a final step of pre-processing,

the region with $96 \times 96\ mm^2$ centered at the pancreatic neck (predicted cut surface during pancreatidoduodenectomy) was cropped from the original images.

Four deep convolutional neural networks (ResNet, DenseNet, ResNeXt, and Inception v3) were utilized to develop the CT-based deep learning models.[11-14] The first convolutional layers of deep convolutional neural networks were modified to have an input channel of 1. A dropout layer and a sigmoid layer were appended to the last fully connected layer of the networks. To reduce the interdependent network elements, the dropout layer randomly ignored the hidden layer nodes in the training process with a probability rate of 0.25.

To train the deep learning models, the training dataset was divided into the training subset (for model development) and the validation subset (for evaluation of models' performance with different hyperparameter values and for the detection of any overfitting that occurred during the training course). Patients who underwent surgery between 2016 and 2017 were randomly separated into the training (728 patients) and validation (153 patients) subsets. Models were learned by the Adam optimizer with a batch size of 32 and a learning rate of 1e-4. The loss function was binary cross-entropy. A maximum epoch was 300; however, when the loss in the validation set did not decrease for 10 epochs, the training was aborted. The implementation of the models was conducted in Python 3.8 with Pytorch 1.8 with Nvidia GTX 2080 ti.

The gradient-guided class attention maps (Grad-CAM++)[15] overlaid with CT images were generated by averaging each attention map of the deep learning models included in the ensemble models.[16] Two model values (ResNet and Inception v3) were averaged for predicting all POPF, and three model values (ResNet, DenseNet, and ResNeXt) were averaged for predicting CR-POPF.

Ensemble model

Ensemble learning was performed separately for making a preoperative model and a comprehensive model. Machine learning, deep learning, and prior models[17,18] were included by ensemble voting, by using the soft or hard voting method.[19] In hard voting, the output of the ensemble was the proportion of models that predicted the class as positive (i.e., the probability predicted by a model was >0.5); however, in soft voting, the output of the ensemble was the average of probabilities predicted by each model. For each voting method, we searched all possible combinations with grid-search methods. The final ensemble of the models was chosen according to two conditions: (1) highest accuracy in the validation subset and (2) absolute difference <5% between the accuracies of the training and validation subsets to avoid overfitting.

References

1. Kazemi-Bajestani SM, Mazurak VC, Baracos V. Computed tomography-defined muscle and fat wasting are associated with cancer clinical outcomes. *Semin Cell Dev Biol.* 2016;54:2-10.
2. Tewari N, Awad S, Macdonald IA, Lobo DN. A comparison of three methods to assess body composition. *Nutrition.* 2018;47:1-5.
3. Prado CM, Lieffers JR, McCargar LJ, et al. Prevalence and clinical implications of sarcopenic obesity in patients with solid tumours of the respiratory and gastrointestinal tracts: a population-based study. *Lancet Oncol.* 2008;9(7):629-635.
4. Martin L, Birdsell L, Macdonald N, et al. Cancer cachexia in the age of obesity: skeletal muscle depletion is a powerful prognostic factor, independent of body mass index. *J Clin Oncol.* 2013;31(12):1539-1547.
5. Arik SÖ, Pfister T. Tabnet: Attentive interpretable tabular learning. Paper presented at: Proceedings of the AAAI Conference on Artificial Intelligence2021.
6. Buitinck L, Louppe G, Blondel M, et al. API design for machine learning software: experiences from the scikit-learn project. 2013.
7. Manaswi NK. Understanding and working with Keras. In: *Deep Learning with Applications Using Python.* Springer; 2018:31-43.
8. Larue RT, van Timmeren JE, de Jong EE, et al. Influence of gray level discretization on radiomic feature stability for different CT scanners, tube currents and slice thicknesses: a comprehensive phantom study. *Acta oncologica.* 2017;56(11):1544-1553.
9. Meijering EHW. Spline interpolation in medical imaging: Comparison with other convolution-based approaches. Paper presented at: 10th European Signal Processing Conference2000.
10. Nalepa J, Marcinkiewicz M, Kawulok M. Data augmentation for brain-tumor segmentation: a review. *Front Comput Neurosci.* 2019;13:83.
11. Xie S, Girshick R, Dollár P, Tu Z, He K. Aggregated residual transformations for deep neural networks. Paper presented at: Proceedings of the IEEE conference on computer vision and pattern recognition2017.
12. He K, Zhang X, Ren S, Sun J. Deep residual learning for image recognition. Paper presented at: Proceedings of the IEEE conference on computer vision and pattern recognition2016.
13. Szegedy C, Vanhoucke V, Ioffe S, Shlens J, Wojna Z. Rethinking the inception architecture for computer vision. Paper presented at: Proceedings of the IEEE conference on computer vision and pattern recognition2016.
14. Huang G, Liu Z, Van Der Maaten L, Weinberger KQ. Densely connected convolutional networks. Paper presented at: Proceedings of the IEEE conference on computer vision and pattern recognition2017.
15. Chattopadhay A, Sarkar A, Howlader P, Balasubramanian VN. Grad-cam++: Generalized gradient-based visual explanations for deep convolutional networks. Paper presented at: 2018 IEEE Winter Conference on Applications of Computer Vision (WACV)2018.
16. Rajaraman S, Kim I, Antani SK. Detection and visualization of abnormality in chest radiographs using modality-specific convolutional neural network ensembles. *PeerJ.* 2020;8:e8693.
17. Roberts KJ, Hodson J, Mehrzad H, et al. A preoperative predictive score of pancreatic fistula following pancreatoduodenectomy. *HPB (Oxford).* 2014;16(7):620-628.
18. Mungroop TH, van Rijssen LB, van Klaveren D, et al. Alternative Fistula Risk Score for Pancreatoduodenectomy (a-FRS): Design and International External Validation. *Ann Surg.* 2019;269(5):937-943.
19. Lee H, Yune S, Mansouri M, et al. An explainable deep-learning algorithm for the detection of acute intracranial haemorrhage from small datasets. *Nat Biomed.* 2019;3(3):173-182.
